# Correlation of antinuclear antibody and anti-double-stranded DNA antibody with clinical response to infliximab in patients with rheumatoid arthritis: a retrospective clinical study

**DOI:** 10.1186/ar3546

**Published:** 2011-12-22

**Authors:** Naoichiro Yukawa, Takao Fujii, Seiko Kondo-Ishikawa, Hajime Yoshifuji, Daisuke Kawabata, Takaki Nojima, Koichiro Ohmura, Takashi Usui, Tsuneyo Mimori

**Affiliations:** 1 Department of Rheumatology and Clinical Immunology, Graduate School of Medicine, Kyoto University, 54 Shogoin Kawahara-cho, Sakyo-ku, Kyoto 606-8507, Japan

## Abstract

**Introduction:**

The induction of antinuclear antibodies (ANAs) or anti-double-stranded (ds) -DNA antibodies (Abs) after infliximab (IFX) therapy in rheumatoid arthritis (RA) is a well-known phenomenon, but the correlation of such Abs with the clinical response to IFX has not yet been determined. The aims of this retrospective observational study were to examine the prevalence of positive ANA and anti-ds-DNA Abs before and after IFX therapy in patients with RA and to investigate whether an increased titer of such Abs is associated with the clinical efficacy of IFX.

**Methods:**

One hundred eleven RA patients who had received IFX were studied. ANA (indirect immunofluorescence with HEp-2 cells) and anti-ds-DNA Abs (Farr assay) results were examined before and after IFX therapy.

**Results:**

The overall clinical response assessed by EULAR response criteria was as follows: good response in 55%, including remission in 38%; moderate response in 18%; and no response (NOR) in 27%. The positivity of ANA (≥ 1:160) and anti-ds-DNA Abs significantly increased from 25% to 40% (*P *= 0.03) and from 3% to 26% (*P *< 0.001) after IFX, respectively. EULAR response differed significantly according to the ANA titer before IFX (*P *= 0.001), and the efficacy of IFX became worse as the ANA titer before starting IFX increased. Furthermore, the differences in the clinical response of the ANA titer before IFX ≤ 1:80 and ≥ 1:160 were significant (good, moderate, and no response were 66%, 9%, and 25% in ≤ 1:80 group versus 26%, 33%, 41% in ≥ 1:160 group, respectively; *P *< 0.001). In 13 patients whose ANA had increased after IFX, 10 showed NOR, only one showed a good response, and none reached remission. These clinical responses were significantly different from ANA no-change patients. In 21 patients with positive anti-ds-DNA Abs after IFX, 16 showed NOR, only two showed a good response, and none reached remission.

**Conclusions:**

The present study suggests that the ANA titer before starting IFX predicts the clinical response to IFX. The increased titers of ANA or anti-ds-DNA Abs after IFX may be useful markers of NOR.

## Introduction

Rheumatoid arthritis (RA) is a chronic, inflammatory disease with the potential to cause substantial joint damage and disability. Tumor necrosis factor (TNF)-α plays a central role in the pathogenesis of RA, as demonstrated by the clinical benefit of anti-TNF-α therapy [1-6]. Infliximab (IFX), a chimeric anti-human TNF-α monoclonal antibody, has enabled great advances in the treatment strategy for RA, resulting in a paradigm shift of RA treatment. Although IFX therapy concomitant with methotrexate (MTX) is effective in the majority of RA patients, some patients have persistent active disease, and others lose efficacy after prolonged treatment [5-7]. However, no useful clinical marker has been established to predict such nonresponse (NOR) to IFX.

The induction of antinuclear antibodies (ANAs) and anti-double stranded (ds)-DNA antibodies (Abs) during IFX therapy is a well-known phenomenon that has already been observed in earlier clinical trials [1-3]. It has been reported that the induction of ANAs is independent of the IFX dose [2,8] and is not modified by concomitant treatment with MTX [9,10], leflunomide, and corticosteroid [8]. Furthermore, the production of ANA is not associated with the clinical response to IFX [11], and even when the development of anti-ds-DNA Abs is observed, onset of lupus-like symptoms is extremely rare [12]. Thus, the significance of the development of such antibodies, including correlations of ANAs and anti-ds-DNA Abs with NOR in RA, has not yet been determined.

Recently, it was reported that the development of ANAs and anti-ds-DNA Abs with anti-TNF therapies may act as a marker of forthcoming treatment failure in patients with psoriasis [13]. Conversely, as in RA patients, it has been reported that ANAs are a predictive factor of infusion reactions during IFX as well as without MTX [14]. On the basis of these findings, the aims of this retrospective observational study were to examine the prevalence of positive ANAs and anti-ds-DNA Abs before and after IFX therapy in patients with RA, and to investigate whether the induction or increased titer of such Abs is associated with the clinical efficacy of IFX.

## Materials and methods

### Patients and administration of infliximab

One hundred eleven Japanese patients with RA, who had started using IFX as the first biologic agent from November 2003 to June 2009 in our hospital, were studied. All the patients had met the 1987 revised criteria of the American College of Rheumatology (ACR) for the classification of RA [15]. IFX concomitant with MTX was given at 0 (initial dose of 3 mg/kg), 2, and 6 weeks, and then every 8 weeks. If the efficacy of IFX was insufficient, we were permitted to increase the dosage up to the full-bottled dose (for example, 150 mg to 200 mg in a patient weighing 50 kg) or to shorten the administration interval up to every 6 weeks from 8 weeks, according to the judgment of the attending physician. Disease activity was assessed by the disease-activity score in 28 joints (DAS28 ESR) [16], and clinical responses to IFX were evaluated with the European League against Rheumatism (EULAR) response criteria [17]. In contrast to primary NOR patients who had never achieved moderate or good response, loss of response (LOR) was defined as DAS28 score returned to NOR according to the EULAR criteria, after having maintained moderate or good response during at least 3 times of administration of IFX. The present study was conducted in compliance with the Declaration of Helsinki and was approved by the Kyoto University Ethics Committee Review Board, and written informed consent was obtained from all patients.

### Determination of study point

Determination of the study point after the IFX therapy is different in each group. In moderate- or good-response patients, the data were collected at a stable point after at least three consecutive administrations of IFX after an achievement of a moderate or good response. In LOR patients, the data were collected within 3 months after LOR was observed. In the IFX withdrawal group (including primary NOR patients), the data were collected just before IFX was discontinued. The intervals between before and after IFX were 6 to 286 weeks (mean, 87 ± 57 weeks), and the total number of IFX administrations was 3 to 38 times (mean, 13 ± 7.3 times), respectively.

### Determination of antinuclear antibody and anti-ds-DNA antibody

ANAs and anti-ds-DNA Abs were examined before and after IFX therapy. ANAs were determined by indirect immunofluorescence with HEp-2 cells, and anti-ds-DNA Abs by the Farr assay (normal, < 6 U/ml).

### Statistical analysis

Statistical analysis was performed with PAWS version 18 software, by using the Fisher Exact test for changes of ANAs and anti-ds-DNA Abs before and after therapy, the Jonckheere-Terpstra trend test for correlations between ANA titers and clinical response to IFX, and the χ^2 ^test for comparison between two groups (including ANA ≤ 1:80 group versus ≥ 1:160 group, and ANA titers in the no-change group versus increased group after therapy, respectively), respectively. A value of *p *< 0.05 was considered significant.

## Results

### Characteristics of the patients and clinical efficacy of IFX

The characteristics of 111 RA patients are shown in Table 1: 82% were female patients; mean age was 51 years; and mean disease duration was 6.6 years at the baseline. Mean DAS28 before IFX was 5.37, and MTX was used in all patients at a mean dosage of 8.1 mg/week. Corticosteroids were used in 61%, and the mean dosage of prednisolone (PSL) was 6.2 mg/day. At the study point, the total number of IFX administrations was 3 to 38 times (mean, 13 times). DAS28 had fallen to 3.55 from 5.37, and EULAR responses were as follows: good response in 55%, including remission in 38%; moderate response in 18%; and NOR in 27%, including LOR in 21%, respectively.

**Table 1:** Characteristics of 111 RA patients

Baseline (before starting IFX)	
Female, age (mean ± SD, range)	91/111 (82%), 51.6 ± 13.3 years (21 ~ 80)
Disease duration (mean ± SD, range)	6.6 ± 6.4 years (4 months ~ 32 years)
DAS28 (ESR) (mean ± SD, range)	5.37 ± 1.33 (1.71 ~ 8.41)
MTX (mean ± SD, range)	(used in all the patients), 8.1 ± 1.6 mg/week (4 ~ 14 mg)
Corticosteroids users	68/111 patients (61%)
PSL dosage (mean ± SD, range)	6.2 ± 3.2 mg/day (2 ~ 16 mg)
At the study point	

Total number of IFX (mean ± SD, range)	13.0 ± 7.3 times (3 ~ 38)
DAS28(ESR) (mean ± SD, range)	3.55 ± 1.64 (0.54 ~ 7.84)
EULAR response	Good response 55% (including remission 38%)
	Moderate response 18%
	NOR 27% (including LOR 21%)
Discontinuation	45/111 (41%)
Reasons for discontinuation	Remission, eight; NOR, 21; adverse events, 14;
	financial reasons, two

IFX, infliximab; LOR, loss of response; MTX, methotrexate; NOR, no response; PSL, prednisolone.

### Positivity of ANA and anti-ds-DNA Abs before and after therapy

**Figure 1:**
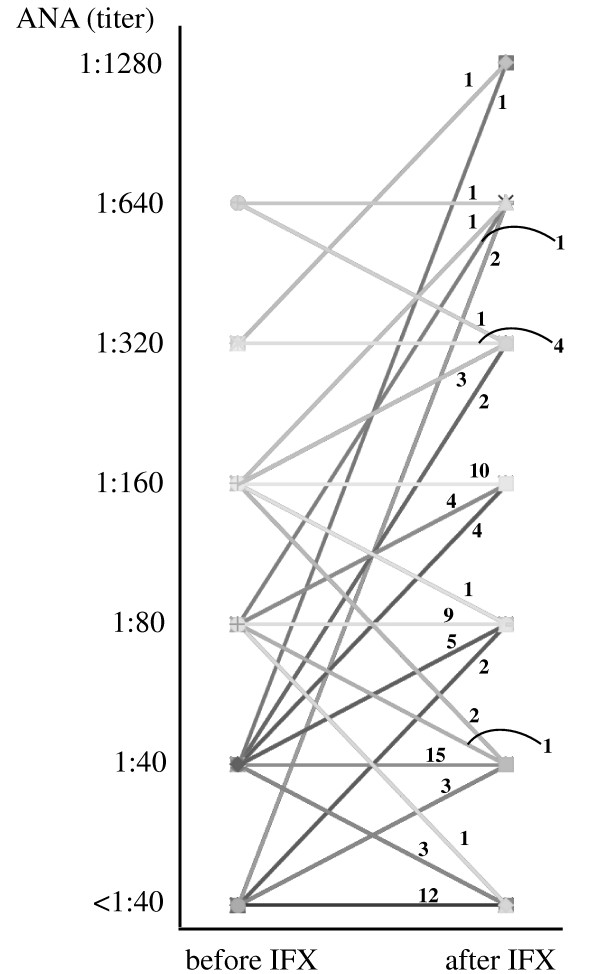
**Changes of ANA titer between before and after IFX**. The numbers in the graph indicate the numbers of patients. IFX, infliximab; ANAs, antinuclear antibodies.

The prevalence of positive ANA (≥ 1:40) did not change before and after IFX (78% to 82%), but with ANA ≥ 1:160, the prevalence significantly increased from 25% to 40% (Table 2; *P *= 0.03, Fisher Exact test). Furthermore, the positivity of anti-ds-DNA Abs significantly increased from 3% to 26% (*P *< 0.001; Fisher Exact test). The changes of ANA titer between before and after IFX are shown in Figure 1.

**Table 2:** Positivity of ANAs and anti-DNA Abs before and after IFX

		Before IFX	After IFX	***P *value**^**a**^
ANA	≥ 1:40	83/106	77/94	NS
		78%	82%	
	
	≥ 1:160	27/106	38/94	0.03
		25%	40%	

anti-DNA	≥ 6 U/ml	3/93	21/80	< 0.001
		3%	26%	

ANA, antinuclear antibodies; anti-DNAs, anti-ds-DNA antibodies; IFX, infliximab; NS, not significant. ^a^Analyzed with the Fisher Exact test.

### Correlation between ANA titer and clinical response to IFX

**Figure 2:**
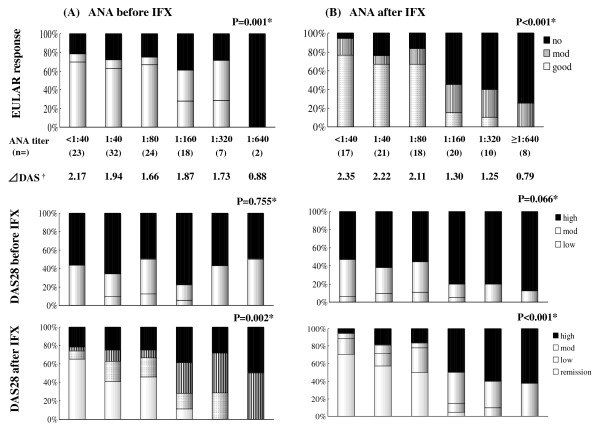
**Correlation between ANA titer and clinical response to IFX**. **(a) **Correlations between ANAs before starting therapy with IFX and clinical response to IFX. EULAR response (upper) and DAS28 after IFX (lower) were significantly different by ANA titer before IFX (*P *= 0.001 and 0.002, respectively, by Jonckheere-Terpstra trend test). The efficacy of IFX became worse as the ANA titer increased before starting IFX. **(b) **These clinical responses to IFX were analyzed with ANAs after starting IFX. The differences became more marked (*P *< 0.001 in both EULAR response (upper) and DAS 28 after IFX (lower), by the Jonckheere-Terpstra trend test). *Analyzed with the Jonckheere-Terpstra trend test. ^†^Mean changes of DAS28 score before and after IFX.

Next, we studied the correlation between the ANA status before starting therapy with IFX and the clinical response to IFX. EULAR response (Figure 2(a), upper) and DAS28 after IFX (Figure 2(a), lower) were significantly different by ANA titer before starting IFX (*P *= 0.001 and 0.002, respectively, by the Jonckheere-Terpstra trend test), and the efficacy of IFX became worse if the ANA titer before IFX increased. No correlation was found between ANA titer before IFX and DAS28 before IFX (Figure 2(a), middle). In addition, clinical responses seemed to be divided into two groups between the ANA titer before IFX with ≤ 1:80 and ≥ 1:160 in this figure. As shown in Table 3, the differences in the EULAR response between these two groups were significant (*P *< 0.001, χ^2 ^test). Furthermore, when these clinical responses to IFX were analyzed by the ANA titers after starting IFX, such tendencies became more marked, as shown in Figure 1(b) (*P *< 0.001 in both EULAR response (upper) and DAS 28 after IFX (lower), by the Jonckheere-Terpstra trend test). In the comparison of the ANA titer before IFX and the EULAR response, a significant difference in the ANA titer after IFX ≤ 1:80 and ≥ 1:160 was observed.

**Table 3:** Comparison of clinical response between ANA titer ≤ 1:80 and ≥ 1:160

	ANA titer					
	
	Before IFX			After IFX		
	
EULAR	≤ 1:80	≥ 1:160	*P *value^a^	≤ 1:80	≥ 1:160	*P *value^a^
response	(*n *= 79)	(*n *= 27)		(*n *= 56)	(*n *= 38)	
Good	66%	26%	< 0.001	77%	11%	< 0.001
(remission)	(16%)	(7%)	< 0.001	(59%)	(3%)	< 0.001
Moderate	9%	33%	< 0.001	7%	29%	< 0.001
No	25%	41%	< 0.001	16%	60%	< 0.001

ANA, antinuclear antibodies; IFX, infliximab; ^a^Analyzed with a χ^2 ^test.

### Clinical response of patients with increased ANA titer after IFX

Based on these results, we next examined patients whose ANAs had increased by two or more dilution levels (for example, ANA titer from < 1:40 to 1:80, from 1:80 to 1:320, and so on) after treatment with IFX (Table 4). Among these 13 patients, 10 showed NOR, only one showed a good response, and none reached remission. These clinical responses were significantly different from those of the ANA no-change patients (Table 5).

**Table 4:** Clinical response of the 13 patients with increased ANA titer after IFX

	ANA titer		EULAR response
		
	Before	After	
1	< 40	80	Good
2	< 40	80	No
3	< 40	640	No
4	< 40	640	No
5	40	160	No
6	40	160	No
7	40	160	Moderate
8	40	320	No
9	40	320	No
10	40	1,280	No
11	80	640	Moderate
12	160	640	No
13	320	1,280	No

ANAs, antinuclear antibodies; IFX, infliximab.

**Table 5:** Comparison of clinical response between ANA titer increased and no-change patients

	ANA after IFX		
		
EULAR	Increased	No change	***P *value**^**a**^
response	(*n *= 13)	(*n *= 76)	
Good	8%	54%	0.001
(Remission)	(0)	(42%)	0.001
Moderate	15%	20%	0.001
No	77%	26%	0.001

ANAs, antinuclear antibodies; IFX, infliximab.

**^a^**Analyzed with χ^2 ^test.

### Clinical response of patients with positive anti-ds-DNA antibodies after IFX

The characteristics of 21 patients with positive anti-ds-DNA antibodies after IFX are shown in Table 6. Sixteen of 21 patients showed NOR, only two showed a good response, and none reached remission. Interestingly, the second biologic agents, including other TNF antagonists switched from IFX, were all effective in these patients. Furthermore, three patients' clinical responses to IFX were restored by 20-mg PSL before each IFX infusion.

**Table 6:** Twenty-one patients with positive anti-ds-DNA Abs after IFX

		anti-DNA		ANA					
						
	age/sex	before	after	before	after	EULAR response	symptoms	IFX	following biologics
1	51/F	< 2	300	40	320	No		Discontinue	ADA- > TOC*^4^
2	41/F	6	300	320	320	No		Discontinue	ETN
3	45/F	51	300	160	320	No	SLE like*^1^	Discontinue	ETN
4	32/F	NA	52	320	1280	No			
5	31/F	5	42	80	160	No	SLE like*^2^	Discontinue	ETN
6	36/F	5	38	40	1280	No- > good		PSL 20 mg iv*^3^	
7	43/F	4	26	160	640	No		Discontinue	TOC
8	56/F	3	26	80	160	No- > good		PSL 20 mg iv*^3^	
9	62/M	21	25	80	80	Good	PCP	Discontinue	TOC
10	65/F	2	23	160	160	No		Discontinue	TOC- > ETN*^5^
11	52/F	NA	22	NA	160	No			
12	56/M	< 2	20	320	320	No	allergy	Discontinue	ETN
13	33/F	5	19	40	80	No			
14	51/F	< 2	13	40	80	Mod			
15	56/M	< 2	11	160	160	No- > mod		PSL 20 mg iv*^3^	
16	75/F	< 2	11	< 40	320	No		Discontinue	ETN
17	58/F	11	11	80	640	Mod			
18	30/M	< 2	10	80	80	No		Discontinue	ETN
19	35/F	< 2	10	80	80	Good			
20	40/F	3	8	80	160	No		Discontinue	ETN
21	59/F	NA	7	NA	640	Mod			

anti-DNA, anti-ds-DNA antibodies; ANA, antinuclear antibodies; IFX, infliximab; SLE, systemic lupus erythematosus; PCP; pneumocystis jirovecii pneumonia; PSL, prednisolone; iv, intravenous injection; ADA, adalimumab; ETN, etanercept; TOC, tocilizumab; *1, leukocytopenia and alopecia; *2, erythema; *3, premedication of each IFX administration; *4, ADA was also getting no response and successful swiched to TOC; *5, TOC ineffective, and ETN effective

## Discussion

In this study, we found that the high titer of ANAs (≥ 1:160) significantly increased from 25% to 40% in RA patients after using IFX, and the positivity of anti-ds-DNA Abs was also significantly increased from 3% to 26%. These results are similar to those of previous reports. Whereas the induction of ANAs and anti-ds-DNA Abs during IFX therapy is a well-known phenomenon, the mechanisms of autoantibody production are poorly understood, and their clinical significance is unknown. Several reasons for the production of ANAs and anti-ds-DNA Abs have been speculated on.

First, the direct effect of a decreased serum TNF level caused by a TNF blocker is considered. It has been reported that low levels of TNF-α may promote SLE in predisposed mice and that the treatment of (NZB/NZW) F_1 _mice with TNF-α ameliorates nephritis [18], and low TNF-α expression may be implicated also in human SLE patients [19]. Thus the blockade of TNF-α itself may favor a lupus-like autoimmunity phenomenon.

Second, anti-TNF treatment also reduces CRP levels, and CRP is known to help clear nuclear material after apoptosis. Low CRP levels would result in the prolonged exposure of nuclear material and hence further increase the chance of antibody formation [20,21]; however, serum CRP levels can be reduced not only by anti-TNF treatment but also by other antirheumatic drugs, including MTX and corticosteroids, and actually do not differ between patients with and without TNF blockade induced by autoantibodies [22]. Thus, these phenomena alone are not sufficient to explain the production of ANAs or anti-ds-DNA Abs. Moreover, because the reduction of serum TNF levels is caused by etanercept (ETN) and adalimumab (ADA), as well as IFX, it is difficult to explain the differences in the positivity of ANAs after treatment between IFX and ETN and the successful treatment of switching from IFX to ETN in patients with positive anti-ds-DNA Abs.

As already is known, IFX inhibits not only soluble TNF-α but also transmembrane TNF (tmTNF)-α; the binding of IFX to tmTNF-α may provoke apoptotic cell death with the expression of autoantigens that could trigger the development of anti-ds-DNA antibodies [9]. In contrast, ETN binds mainly soluble TNF-α; the ability of IFX to bind tmTNF-α may explain why IFX but not ETN induces the apoptosis of monocytes and T lymphocytes in Crohn disease [23]. A recent article demonstrated that all three anti-TNF drugs were able to bind tmTNF-α exposed by Jurkat cells, a human lymphoblastoid cell line, and to induce similar antibody-dependent cell-mediated cytotoxicity; in contrast, complement-dependent cytotoxicity was more pronounced with anti-TNF-α monoclonal antibodies in comparison with ETN [24]. These data suggest that ETN may be less effective than both IFX and ADA in the elimination of tmTNF-α-expressing cells. The partially different mechanism of TNF-α inhibition between ETN and anti-TNF-α monoclonal antibodies may explain the lesser generation of autoantibodies in patients treated with ETN in comparison with IFX, as well as the clinical efficacy of IFX and ADA but not of ETN in the treatment of granulomatous diseases such as Crohn disease and Wegener granulomatosis.

Thus, some reasonable explanations exist for the production of ANAs; however, it is unclear why their formation should be associated with the clinical response to IFX. Recently, it was shown that ANAs are a predictive factor of infusion reactions during IFX as well as without MTX in RA patients [14]. Furthermore, it was reported that the development of ANA and anti-ds-DNA Abs in anti-TNF therapies may act as a marker of forthcoming treatment failure in patients with psoriasis [13]. To our knowledge, the present study is the first report to clarify the correlation of ANAs and anti-ds-DNA Abs with the efficacy of IFX in RA patients. Moreover, surprisingly, the clinical response to IFX differed by the ANA titer before IFX, and the predictive value of the baseline ANA titer of ≤ 1:80 and ≥ 1:160 for the efficacy of IFX was observed. This result suggests that preexisting autoimmune abnormalities indicated by ANAs may influence the effect of IFX. Conversely, NOR of IFX was more markedly associated with the increase of ANAs or the induction of anti-ds-DNA Abs. Furthermore, in NOR patients, switching to second TNF antagonists and premedication of PSL without discontinuation of IFX were both effective, suggesting that IFX-induced autoimmune responses may affect the efficacy of IFX.

The mechanisms underlying treatment failure in RA patients treated with IFX have not been entirely clarified; however, one important factor may be the development of anti-drug antibody, or human anti-chimeric antibody (HACA) [1,2]. Because these antibodies cannot be routinely measured in the clinical setting, currently no clinically accessible markers of forthcoming treatment failure are available. Although HACA could not be determined in all patients in this study, we could measure serum HACA in three patients who were successfully restored from NOR by premedication with PSL without discontinuation of IFX, and HACA was negative, at least in these three patients.

Recently, Takeuchi *et al*. [7] reported that the clinical efficacy of IFX was correlated with the trough serum IFX level in a prospective randomized control trial (RISING study). The authors speculated that anti-IFX antibody may be an important factor influencing the efficacy of IFX by increasing the serum clearance of IFX; however, anti-IFX antibody and autoantibodies including ANAs were not measured in the RISING study. Although the IFX trough level was also not measured in our study, ANAs and anti-ds-DNA Abs observed in the NOR patients in our study seemed to be indicators of the immunologic response to the decreased clinical efficacy of IFX. Recently, Hoffmann *et al*. [25] reported that ANAs and anti-ds-DNA Abs in psoriasis patients are predictors for LOR and anti-IFX antibody induction [25], and these data strongly supported our speculation. To confirm this speculation, a large-scale clinical study in RA patients is necessary to examine the correlations between the clinical efficacy of IFX and various factors, including the trough serum IFX level, anti-IFX antibody, and autoantibodies (including ANAs and anti-ds-DNA Abs).

One important limitation of this study was that the approved dosage of IFX and MTX in Japan during the study period was only 3 mg

kg every 8 weeks (IFX) and 8 mg/week (MTX), respectively. This approved dosage of IFX and MTX in Japan is much lower than that in Western countries, and it may result in insufficient clinical efficacy of IFX or suppression of immune responses to IFX. We therefore consider that it is necessary to research the production of ANAs and anti-ds-DNA Abs and their correlation with the clinical efficacy of IFX with a sufficient dosage of IFX and MTX.

Recently, several prognostic markers for the efficacy of IFX, including plasma platelet factor 4 [26] and the gene or mRNA profile in peripheral blood cells [27,28], have been reported; however, the measurement of these markers is complicated and commercially unavailable, and the prognostic value remains insufficient. Regarding these points, ANA and anti-ds DNA Abs are routine laboratory tests and can be measured easily and simply in daily clinical practice. In addition, IFX is the only drug showing clinical evidence of the possibility of biologics-free remission and even drug-free remission [29,30] among the several TNF antagonists, so it is necessary to establish prognostic markers of IFX efficacy, especially clinical remission.

## Conclusions

The present study suggests that the ANA titer before starting IFX predicts the clinical response to IFX. Moreover, increased titers of ANAs or the development of anti-ds-DNA Abs after IFX may be useful markers of NOR. Large-scale prospective studies are required to assess the importance of this observation.

## Abbreviations

Abs: antibodies; ACR: American College of Rheumatology; ADA: adalimumab; ANAs: antinuclear antibodies; ds: double stranded; ETN: etanercept; EULAR: European League against Rheumatism; HACA: human anti-chimeric antibody; IFX: infliximab; LOR: loss of response; MTX: methotrexate; NOR: no response; PSL: prednisolone; RA: rheumatoid arthritis; SLE: systemic lupus erythematosus; TNF: tumor necrosis factor; tmTNF: transmembrane TNF.

## Competing interests

The authors declare that they have no competing interests.

## Authors' contributions

NY drafted the manuscript and performed the statistical analysis. NY and TF designed the study. NY, TF, SKI, HY, DK, TN, KO, TU, and TM collected the clinical data. NY, TF, HY, DK, TN, KO, TU, and TM enrolled patients for the study. TM supervised the study design and helped to draft the manuscript. All authors read and approved the final manuscript.
